# Risk and space: modelling the accessibility of stroke centers using day- & nighttime population distribution and different transportation scenarios

**DOI:** 10.1186/s12942-021-00284-y

**Published:** 2021-06-29

**Authors:** S. Rauch, H. Taubenböck, C. Knopp, J. Rauh

**Affiliations:** 1 Institute for Geography and Geology, Julius-Maximilians-Universitat Würzburg, 97074 Würzburg, Germany; 2 German Aerospace Center (DLR), German Remote Sensing Data Center (DFD), Oberpfaffenhofen, 82234 Wessling, Germany

**Keywords:** Accessibility analysis, High resolution population data, Public health

## Abstract

**Purpose:**

Rapid accessibility of (intensive) medical care can make the difference between life and death. Initial care in case of strokes is highly dependent on the location of the patient and the traffic situation for supply vehicles. In this methodologically oriented paper we want to determine the inequivalence of the risks in this respect.

**Methods:**

Using GIS we calculate the driving time between Stroke Units in the district of Münster, Germany for the population distribution at day- & nighttime. Eight different speed scenarios are considered. In order to gain the highest possible spatial resolution, we disaggregate reported population counts from administrative units with respect to a variety of factors onto building level.

**Results:**

The overall accessibility of urban areas is better than in less urban districts using the base scenario. In that scenario 6.5% of the population at daytime and 6.8% at nighttime cannot be reached within a 30-min limit for the first care. Assuming a worse traffic situation, which is realistic at daytime, 18.1% of the population fail the proposed limit.

**Conclusions:**

In general, we reveal inequivalence of the risks in case of a stroke depending on locations and times of the day. The ability to drive at high average speeds is a crucial factor in emergency care. Further important factors are the different population distribution at day and night and the locations of health care facilities. With the increasing centralization of hospital locations, rural residents in particular will face a worse accessibility situation.

**Supplementary Information:**

The online version contains supplementary material available at 10.1186/s12942-021-00284-y.

## Introduction

Stroke as a suddenly occurring severe circulatory disorder of the brain due to cerebral infarction or cerebral hemorrhage is one of the most common neurological diseases (Kolominsky-Rabas/Heuschmann [29]: 658; [3]: 10). It is also one of the main causes of disability and invalidity in adulthood (Busch/Kuhnert [15]: 71, Stahmeyer et al. [52]: 711). Stroke is the second leading cause of death worldwide: Health data of the WHO [65] display for the year 2016 5.78 million deaths worldwide and a crude death rate of 77 per 100.000 inhabitants. The German Federal Statistical Office [58] also lists stroke among the most frequent causes of death in Germany. According to this, 7302 women and 4,722 men died in 2018 of a stroke,this corresponds to a share of 1.3% (female: 1.5%; male: 1.0%) of all causes of death [58]. Despite demographic ageing the number of deaths with stroke as the cause of death has steadily decreased in recent years (2002: 39,433; 2006: 28,566, 2010: 23,675, 2014: 16,753 [54, 55, 56, 57].

There are numerous reasons for this decline of deaths, but it may be assumed that the establishment of a network of specialized care facilities in Germany (“stroke units”) contributed to this development. These stroke units are defined as specialized care centers with appropriate equipment for intensive care and monitoring of affected patients (Hacke and Schuster [26]: 520). The treatment concept includes both, the acute treatment of stroke patients and the treatment of as well as early rehabilitation efforts (Ringelstein and Busse [48]: 7).

Another important aspect for the care of acute stroke patients and of Emergency Medical Services (EMS) in general is the organization of medical first aid. There is a correlation between stroke-related mortality and travel time to the nearest stroke unit [1, 9, 10]. In a key issues paper on emergency medical care in the prehospital and clinical phase, (Fischer et al. [20]: 393) make the following recommendations for stroke care: “*A prehospital time of maximum 60 min to transfer the patient to the nearest suitable hospital with a certified stroke unit is acceptable”.* In addition, Kunz et al. [32] showed that treatment of an ischemic stroke within 60 min provided subsequent functional improvement and improved 3-month survival rate.

**Fig. 1:**
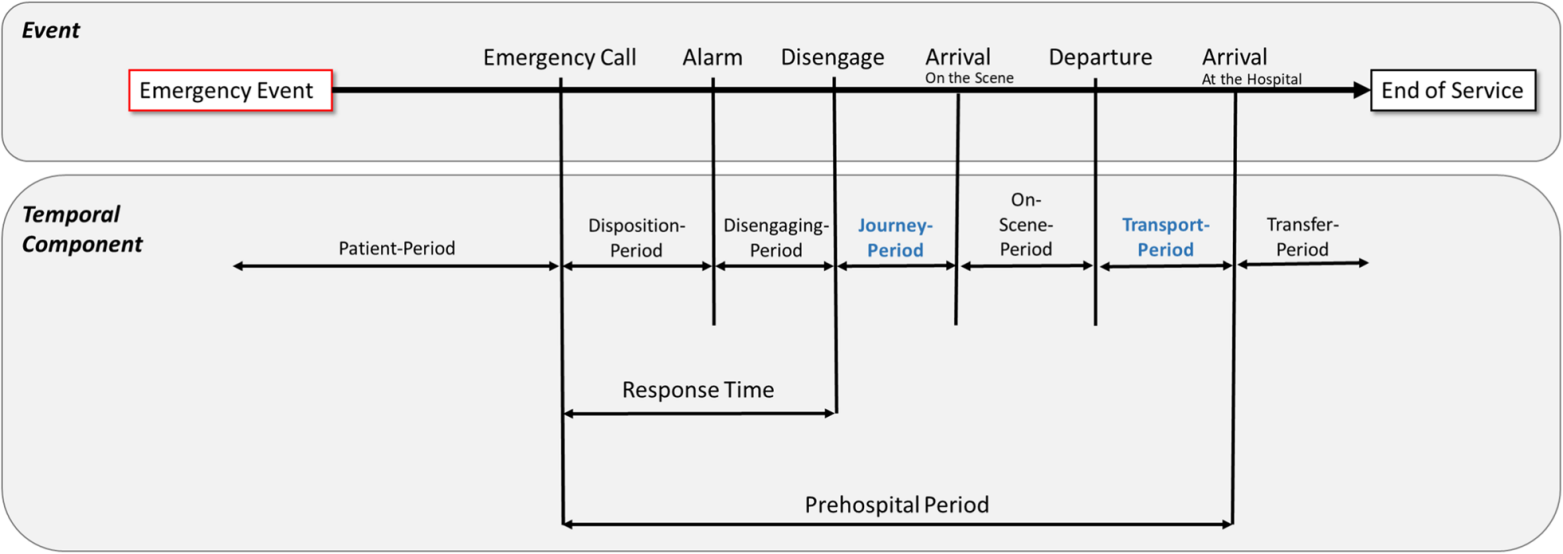
Systematic first aid and transportation time cycle of an emergency event (Fischer et al. [20]: 388)

Figure 1 displays a complete time cycle from an emergency event to treatment in a health care facility systematically (Fischer et al. [20]: 388). In this paper, we focus on the highlighted journey-interval, although the method used is valid for the transport-interval as well.

This interval addresses the concept of accessibility. In a broader sense the concept of accessibility is multi-dimensional [44]. Beside the “spatial dimension such as availability and accessibility […], non-spatial dimensions like affordability, acceptability and accommodation” [43] are highly relevant. In the case of emergency, the rapid accessibility of medical care can decide on the chances of complete recovery and reduction of negative health consequences or even, in the worst case, on survival. The dimension of spatial and temporal accessibility is within the time cycle obviously of great importance for acute stroke care (highlighted blue in Fig. 1). In this study we therefore focus on the concept of “time is brain”, i.e. the temporal accessibility to the nearest facility rather than availability [24, 49]. There are other methods, mostly based on 2SFCA approaches, which also consider availability (Fransen et al. [22], Higgs et al. [27], Chen et al. [16]).

Parvin et al. [43] present the advantages of geographical information systems (GIS) in medical care planning. The specific GIS-methods are very differentiated [43] and have been widely applied in medical supply planning. Still, some challenges with regard to data and methods for modeling accessibility remain:

1. Firstly, the *locations of the patients* play an important role regarding the quality of accessibility to medical facilities. In care planning, addresses of potential patients' residence are used. This is mainly for statistical reasons, since population statistics provide information about address of the place of residence (de jure population). Due to a lack of data, however, aggregated information at administrative levels are generally used. But little is displayed in the statistics about the actual whereabouts of people (de facto population), which varies considerably between day and night.
2. Secondly, in emergencies, *rapid medical first contact* often plays a decisive role. In the prehospital time up (Fig. 1), journey- and transport-periods are the two time-spatial elements, which can be evaluated regardless of the actual emergency event. In order to define certain time zones and to implement them in spatial planning, average speeds need to be assumed. The traffic conditions, however, are strongly dependent on the type of road, speed regulations, (priority) rules for emergency vehicles and the traffic situation [21, 30]. Especially the traffic conditions are subject to very strong fluctuations during the day.
3. The *spatial distribution and density of medical care facilities* is a third essential factor in evaluating the spatial accessibility. In the last decades a mostly economically motivated thinning of the supply networks of general hospitals, specialist clinics, general practitioners, pharmacies etc. has been observed with consequences of increasing travel times and a deterioration in accessibility, especially in peripheral areas [19, 42, 46] anticipate further hospital closures and cuts in the health care system that will also affect the area of stroke care.

The general idea of this study is to present a method that allows to spatially quantify the accessibility of stroke units depending on time of day. Therefore, we take the everyday whereabouts of people and traffic conditions into account. The method used is a straightforward procedure especially suited for macroscale approaches. In this way, the spatial variability of risks shall be mapped. Staying with the "time is brain" concept [49], we use risk as a spatial phenomenon and it is therefore defined by not being reached in time for initial care. Since risk is not spatially static, but varies through the movement of individuals over time, the differences between the locations of people during the day and at night will be determined. This is crucial since not only the daytime population differs from the residential nighttime population, also the potential speeds respectively accessibility is lower during the day [21]. Finally, we also consider the variability of risks by integrating different road transport speeds as a function of traffic volume in general, population density and road type.

### Study area, data and methods

We deal with the following methodical approaches of accessibility analysis using the case study of stroke care:

1. The *day population* (considering the mobility of individuals) differs greatly from the *night population* (residential information) [61]. By using daytime and night-time population distributions at very high spatial resolution, we consider a temporal dimension as well as a more realistic version of spatial population distribution over the day than a simple usage of place of residences. The risk of stroke, as well as the mortality rate, increases with age [14]. Nevertheless, cases also occur in younger age groups. Therefore, within this methodologically oriented study, all potential residences will be considered.
2. Furthermore, *time-based average car speeds* for each class of road are added, in order to reflect daytime and weekly varying traffic load. To evaluate the dynamic effects of different traffic situations on the accessibility situation, we create different scenarios by implementing multiple networks using GIS.
3. Only certified stroke units are considered in our models. There should be no major differences in the provision of services between the stroke units. Analyses that also take the second or third closest center into account are therefore not carried out.

### Study area

We exemplify the spatial risk for a stroke event in the administrative district of Münster, North Rhine-Westphalia, Germany. The district is quite heterogeneous in terms of its settlement structure. It is divided into eight counties (3 city regions, 5 regions with urbanization approach/rural–urban transition^1^). A detailed overview of the population distribution is given in the section entitled Experiment and Results. In order to reduce border problems, in addition to the 15 stroke units located in the area, 23 units from the surrounding districts were considered as well (status 2015).

### Population data

We rely on population projections of the German Federal Statistical Office for 2020, which are based on the 2011 census [56] and the known population dynamics (i.e. migration, births, deaths). Individual addresses of residents are not publicly accessible in full spatial resolution. However, the German Federal Statistical Office provides a summarized INSPIRE-compliant 100 m × 100 m grid data set holding the results from the census 2011 [56]. This corresponds to the population distribution at the place of residence. In our study, however, we refine the model by a temporal and a spatial component: we model the temporal variation of the population distribution for typical day- and nighttime situations and spatially we disaggregate data to a resolution of 20 m. Therefore, we incorporate up-to-date statistical information and very detailed building data to have time-dependent spatial starting points for the accessibility analysis.

We rely on the following demographic data: total population, employees and commuters per economic sector [34, 35, 36], children in schools and day care centers [37, 38] and relative share of care-dependent elderlies [39]. The data is distributed for different administrative units following the Nomenclature of territorial units for statistics (NUTS) developed by Eurostat [18]. The data collected was reported on Local Area Unit (LAU), i.e. municipality level as well as on NUTS-3, i.e. county level and NUTS-2, i.e. district level.

And we rely on cadastral Level-of-Detail-1 (LoD-1) building data provided by the Federal Agency for Cartography and Geodesy (BKG). The dataset provides information on the building ground floor and height as well as on the predominant building usage [2]. This enables to distinguish usage types such as ‘residential’, ‘commercial and industrial’, ‘schools’ and ‘other’. We trained random forest regression models [12] for the different functional types. For example, buildings classified as residential are found to have median storey heights of 3.4 m and a standard deviation of approximately 0.7 m, while buildings that were assigned commercial or service functions have a considerable higher median storey height of 3.8 m in the median as well as a higher standard deviation of 1.2 m, respectively. The retrieved random forest regression models allow to explain 89% of the variance in the test set with a mean absolute error (MAE) of 0.14. This enables us estimating the gross floor area per building.

### Assessment of day- and night-time population

In order to gain the highest possible spatial resolution for the population distribution, we disaggregate the collected statistical information from administrative units (serving as source units) to single building level (serving as target units) following a top-down approach (for reference [4, 23, 67]).

Besides the living space per building, we integrate ancillary information about predominant building usage as well as knowledge about the socio-economic setting within the municipalities including number of employees per economic sector, gross commuting balance per economic sector, number of children and pupils as well as the relative share of care-dependent elderlies.

For daytime modelling the following core assumptions have been made: During the day location of pupils in schools; location of employed persons in the building types linked to the respective economic sector; location of care-dependent persons in elderly home facilities/assisted living; and location of non-employed persons in residential buildings.

For the night situation, our main assumption is the exclusive residence of the population in residential buildings. Following this assumption, one density value is calculated taking the reported total population counts and the respective residential buildings in the LoD-1 building stock into account.

According to the Federal Institute for Occupational Safety and Health [5] overall 80% of the employed population is working between 7 a.m. and 7 p.m. on working days. Approximately 20% of the employees has staggered working hours, i.e. working hours outside 7 a.m. and 7 p.m. In order to keep the model as simple as possible, we define the timeframe for daytime modelling from 7 a.m. to 7 p.m. and vice versa for nighttime.

The disaggregated population is thus—compared to other data sets—available in its temporal variability (day and night) as well as spatially higher resolution on a 20 m × 20 m raster.

**Fig. 2:**
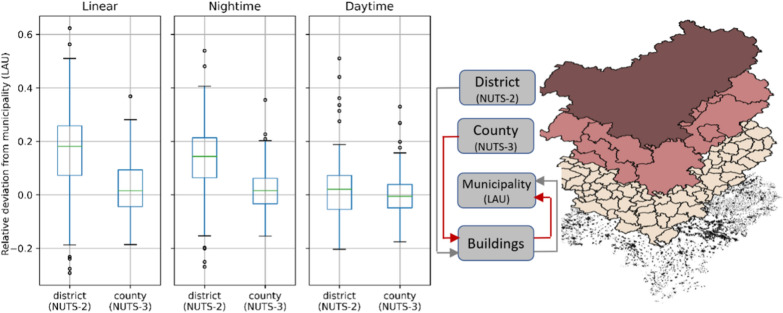
Left: Relative deviation of aggregated modelled numbers from the reported numbers on LAU; Right: Validation approach considering multiple spatial scales, described as NUTS and LAU units. Source data was disaggregated to single building level, aggregated back to municipality (LAU) and compared to the reported numbers

We use a multi-scale approach to validate these data: By carrying out this approach from the NUTS 3 and NUTS 2 level to the building level and then aggregating this result to the independent and previously unused data of the LAU level, validation becomes possible. In addition to the approach described above, we also perform a "linear" estimation without additional information on building use. Thus, the added value of integrating additional socio-demographic and economic data is demonstrated. The deviations of the modelled values are shown as box plots in Fig. 2. The disaggregation of NUTS-2 results in a mean absolute deviation of 17.9% and 9.3% respectively to the figures for LAU in the night and day scenario. The disaggregation of NUTS-3 input data reduces the mean absolute deviation to about 6.4% and 5.9%, respectively.

### Spatial accessiblity

Focusing on accessibility by cars, a street network was used from Open Streetmap data (OSM). Neis et al. [41] mention the dataset is becoming comparable in quality to other geodata from commercial providers (also see [11, 51]) especially in countries with active communities like in Germany. The successful usage of OSM has been demonstrated for accessibility analyses [42, 46, 53, 66]. The influence of one-way streets was also be considered with the help of the data. For each road segment we calculate specific driving speeds as a function (F1) of the maximum speed ($$V_{{max}} )$$, a space-dependent parameter *cfa* (proposed for the study area with 0.85) and its surrounding population density by using the suggested method for OSM data presented by BBSR [6]. The constant value for *k* is either 5000 for highways and highway-like routes or 10,000 for all other routes.

(F1)

$$v = V_{{max}} *cfa*\left( {1 - \frac{{Population~density~within~a~1km~radius}}{k}} \right)$$

The goal of this form of network attribution was to represent traffic jam risk in more densely populated communities and settlement areas, i.e., to simulate a more stressed road network. BBSR [6] and Schwarze/Spiekermann [50] compare this method with FCD and GoogleMaps data. They have concluded that the approach used here when compared to measured travel speeds is within the established range for extreme network conditions (disturbed network, free network) and shows a high correlation to the results of the real-time data. Besides the given infrastructure, this estimated value is the central factor in accessibility analyses. This method results in the base scenario we used for day- & nighttime analyses. The method used here is a straightforward procedure that considers essential influencing factors and is therefore particularly suitable for macroscale approaches. All accessibility models are based on assumptions concerning the network state at a certain time. In reality, there are significant differences in daily and weekly accessibility. The traffic flow is susceptible to disruptions and is sometimes subject to very large fluctuations. Therefore, we introduce further scenarios by reducing (− 30%, − 20%, − 10%) or increasing (+ 10%,  + 20%,  + 30%,  + 40%) the speed for each edge, in order to determine how different daytime mobility might effect the overall accessibility situation. In addition, the respective scenarios (P10–P40) simulate priority rules for emergency vehicles that allow higher speeds on the particular road types in the event of an emergency [45]. Other forms of transport, such as helicopters, are also relevant in the emergency treatment of strokes. However, since their share in first aid is low and the availability and cost of helicopters are problematic [33, 47], the study focuses exclusively on car transport. Table 1 shows the modeled scenarios. The travel time to the closest stroke unit was calculated for each day- and nighttime population point in each scenario. The outcomes of the resulting 16 calculations are discussed below.

**Table 1:** Scenarios by varying velocities

Scenario	Speed change	Purpose
M30 (worst case) M20 M10	Reducing base model speed for every individual road feature by 30% (M30) 20% (M20) 10% (M10)	Simulate the influence of increased traffic volume of different degrees
Base scenario	Base model using method presented by BBSR 2019	Averaged traffic situation
P10 P20 P30 P40 (best case)	Increasing base model speed for every individual road feature by 10% (P10) 20% (P20) 30% (P30) 40% (P40)	Simulate the influence of less traffic volume or better service vehicles of different degrees; Using priority rules for emergency vehicles

## Experiment and results

**Fig. 3:**
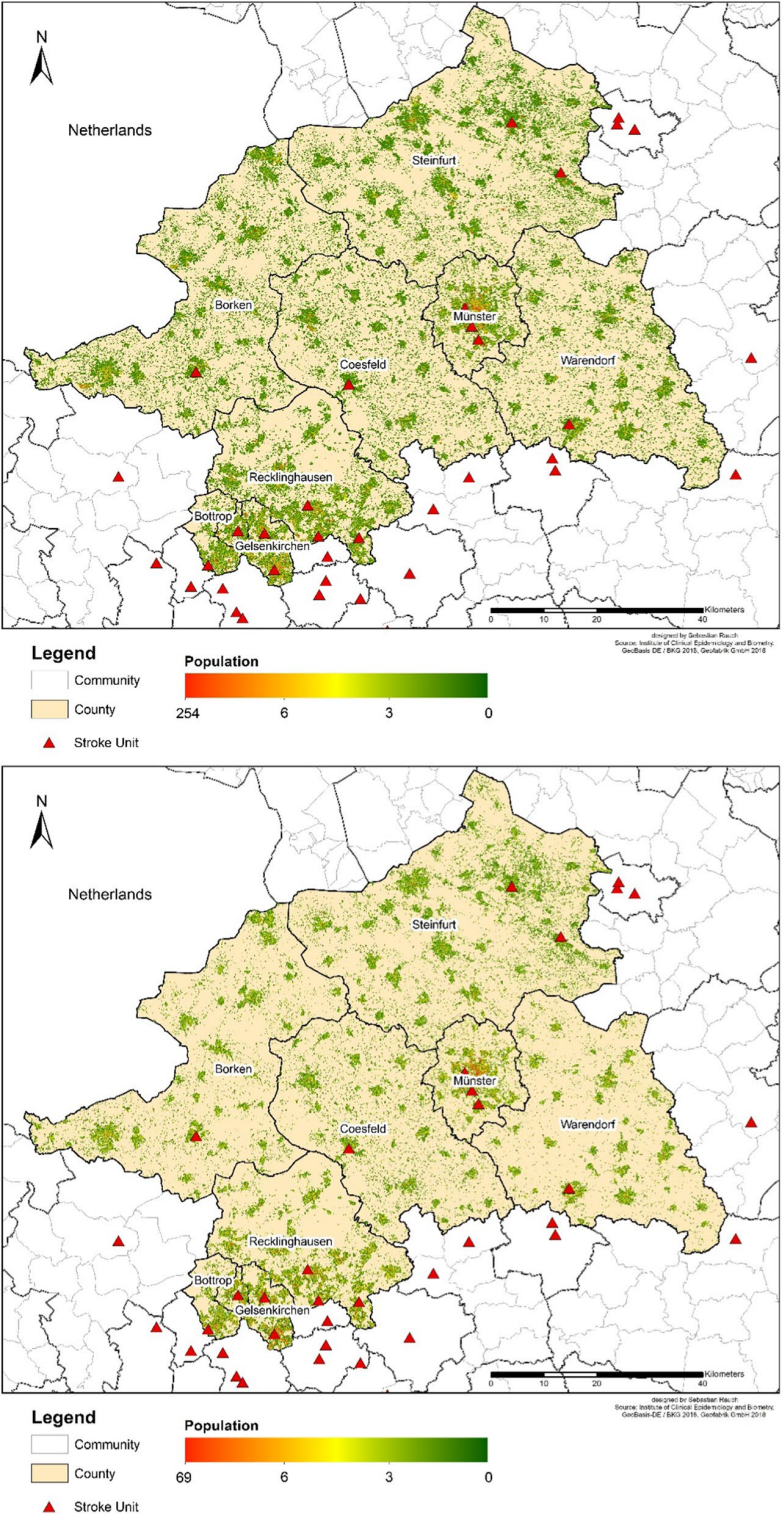
Results of modeled daytime (top) and nighttime (bottom) populations

We focus on three essential elements of spatial supply: *First*, a representation of the accessibility of the day- and nighttime population is carried out. *Secondly*, the effect of different traffic situations is examined and *finally* the supply situation in different settlement categories is observed. Figure 3 shows the modeled population distribution on which the following accessibility analyses are based. An additional high resolution image of the maps helps to visualize the regional differences (see Additional files 1, 2).

The overall modelled daytime population in our research area is 2,527,000, the night population is 2,619,000.

### Focusing day- & night-time population

**Fig. 4:**
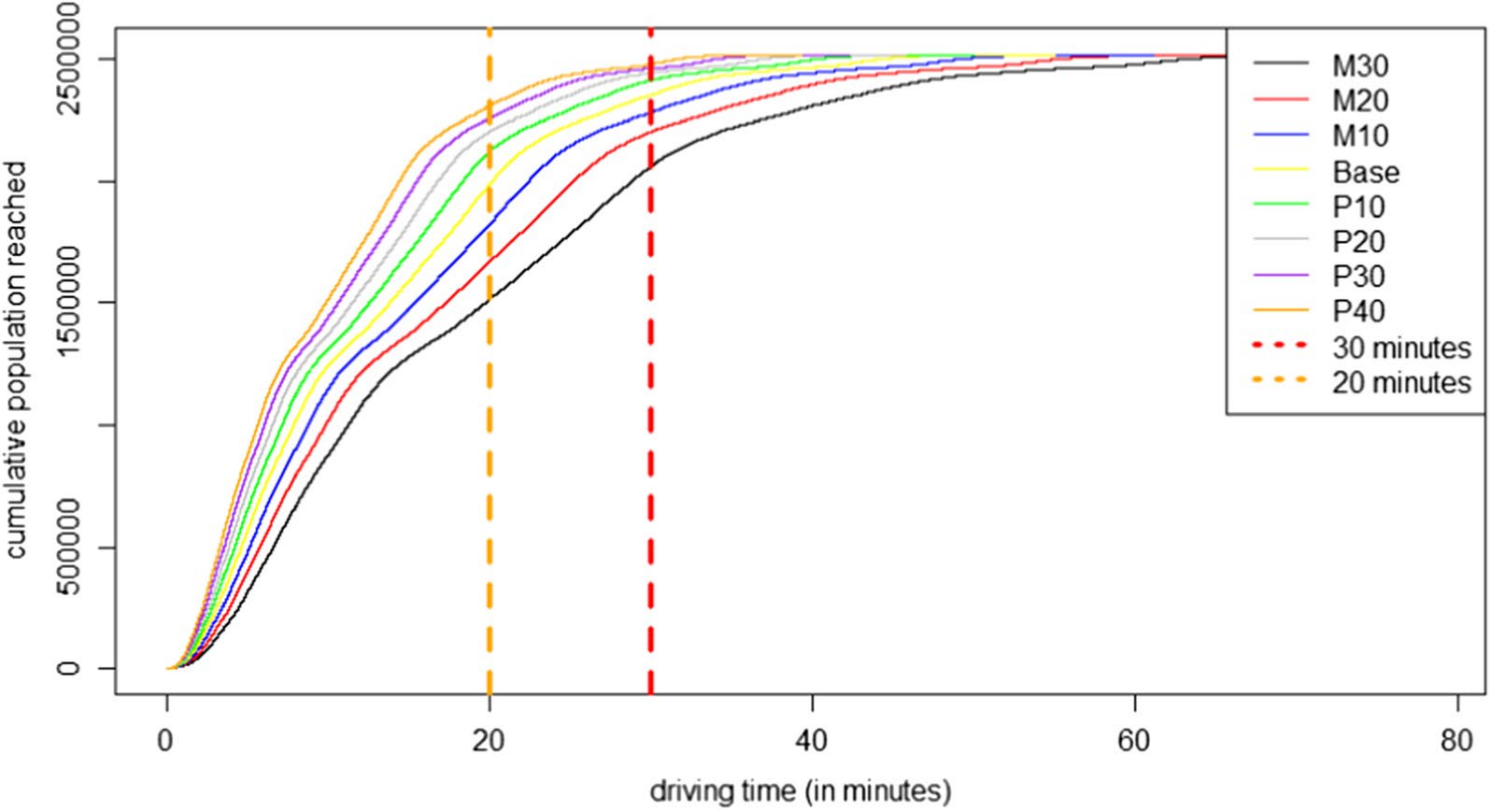
Daytime: Cumulative population by driving time to the nearest stroke unit

**Fig. 5:**
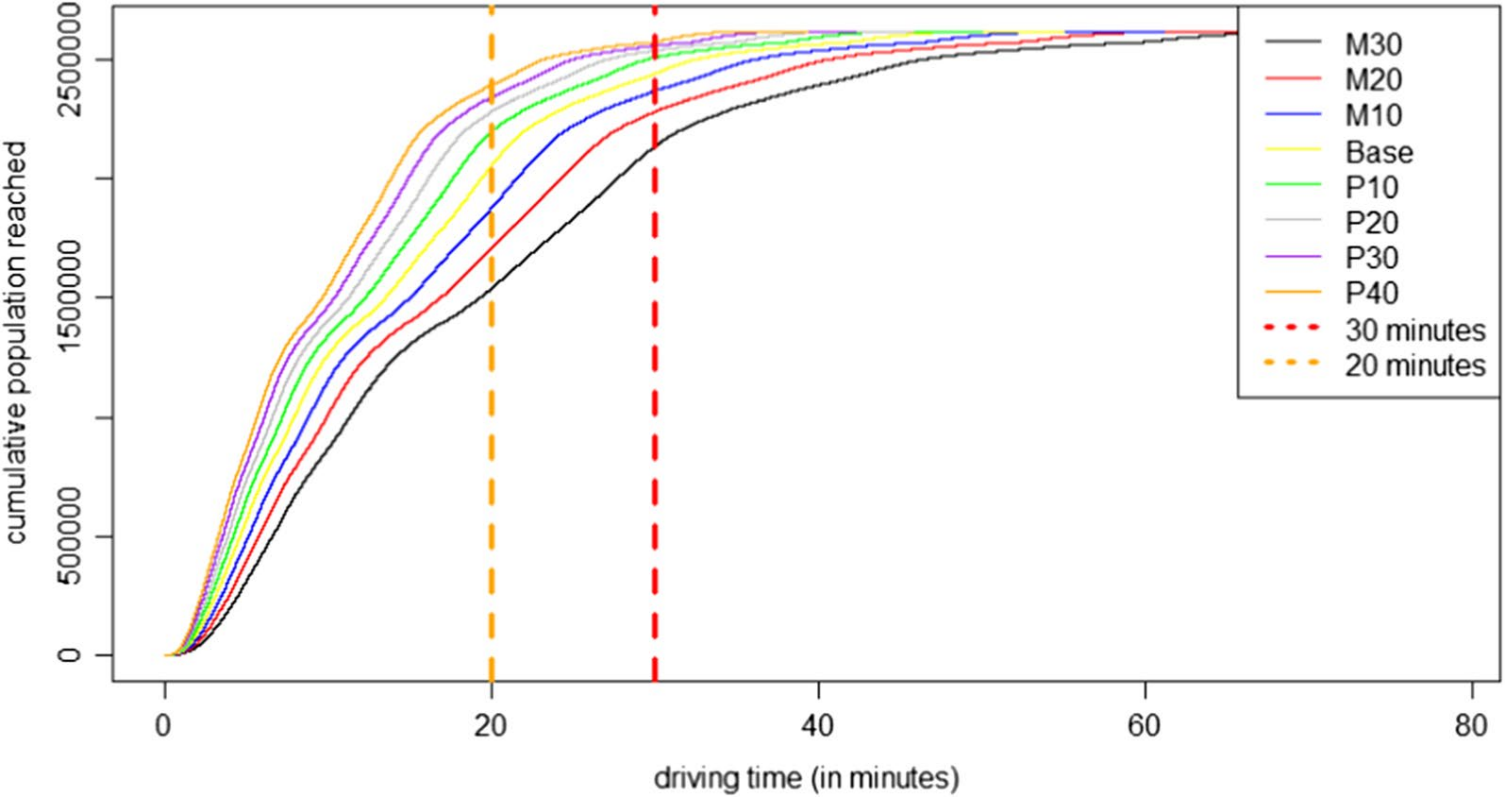
Nighttime: Cumulative population by driving time to the nearest stroke unit

Using the base scenario, we find 6.5% of the people (day) and 6.8% (night) are not within a 30-min driving distance towards a stroke unit. The 30-min limit for first aid in case of an emergency was proposed by a joint initiative of the Institute of Emergency Medicine and Medical Management, the University Hospital of Munich, and the Association of Southwest German Emergency Physicians [13]. For further differentiation, the 20-min interval was also considered. The number of people not reached for first care (journey-interval) within 20 min increases to 21.0% (day) and 21.5% (night). We find the mean travel time by day is 14.6 min and 13.8 min by night. Figures 4 and 5 depict the cumulated reached population for each model. The graphs are similar due to the macroscale view. The only slight differences in accessibility are caused by the fact that the study area is quite urban. Therefore, many population points are spatially equal (daytime population 1,638,487 pixels, nighttime population 1,156,200 pixels, of which 1,035,087 are spatially equal). However, 669,000 modeled individuals reside at night in locations where no one is found during the day. The share of people modeled in the other locations varies, in some cases significantly. There are two slope changes in the graphs. The first change, at around 1.4 million people, is due to people living in high dense urban areas. The accessibility situation becomes worse for less central living and working people. In addition, the two figures (Figs. 4 & 5) show the gap between the M30 scenario and the P-models, especially for a 20-min journey period. Table 2 presents the values and attributes of each scenario.

**Table 2:** Results of the accessibility analyses in the region of Muenster (in minutes) (N(Day) = 2,527,000; N(Night) = 2,619,000)

	Quartile 1	Median	Quartile 3	Maximum	Share of people not reached within 30 minutes	Share of people not reached within 20 minutes
Day						
M30	11.1	20.8	30.2	78.5	18.1	39.8
M20	9.7	18.2	26.5	68.7	12.4	33.6
M10	8.6	16.2	23.5	61.1	9.3	27.4
Base	7.7	14.6	21.2	54.9	6.5	21.0
P10	7.0	13.3	19.2	49.9	4.1	15.7
P20	6.5	12.2	17.6	45.8	2.9	12.4
P30	5.9	11.2	16.3	42.3	2.2	10.3
P40	5.5	10.4	15.1	39.3	1.5	8.2
Night						
M30	10.1	19.7	29.3	78.5	18.4	41.2
M20	8.8	17.2	25.6	68.7	12.8	34.6
M10	7.9	15.3	22.8	61.1	9.5	28.2
Base	7.1	13.8	20.5	54.9	6.8	21.5
P10	6.4	12.5	18.6	49.9	4.2	15.9
P20	5.9	11.5	17.1	45.8	3.0	12.8
P30	5.4	10.6	15.8	42.3	2.2	10.6
P40	5.1	9.9	14.6	39.2	1.6	8.6

**Fig. 6:**
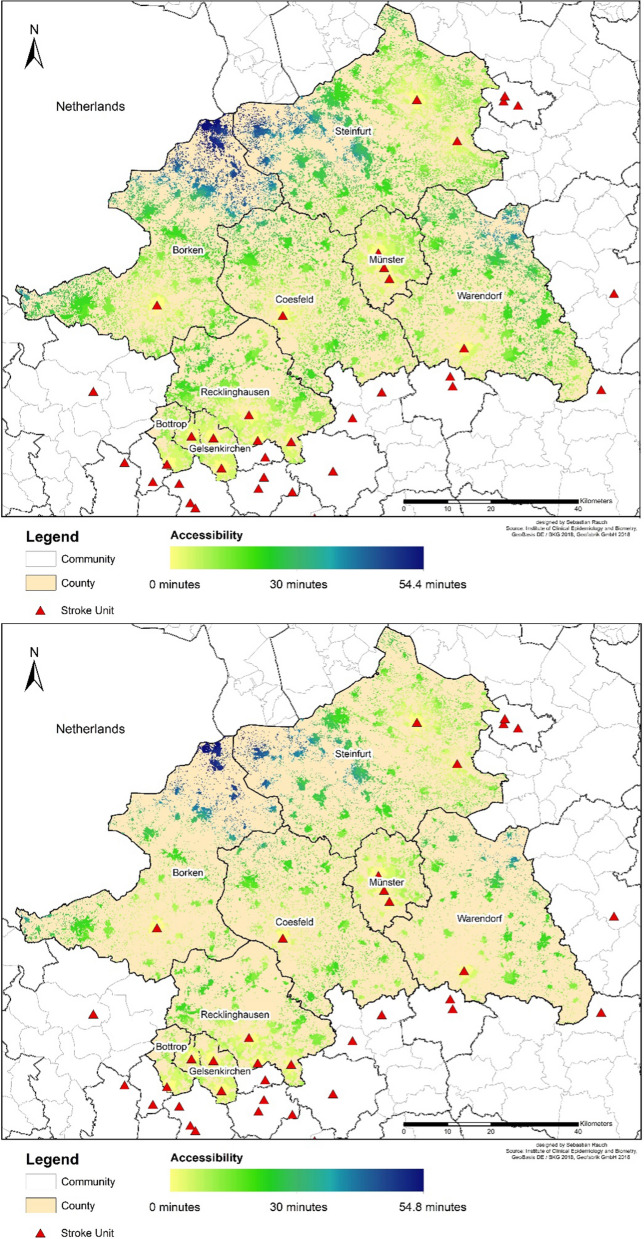
Accessibility of stroke units: Day- (top) and nighttime (bottom) accessibility of stroke units using the base speed model

The maps (Fig. 6) show the positions of the existing stroke units used in the model, and the varying accessibility situations at day- (top) compared to nighttime (bottom). In addition to the poorer supply situation in peripheral areas, it is also evident that the night population is less centrally allocated.

### Focusing different traffic situations

**Fig. 7:**
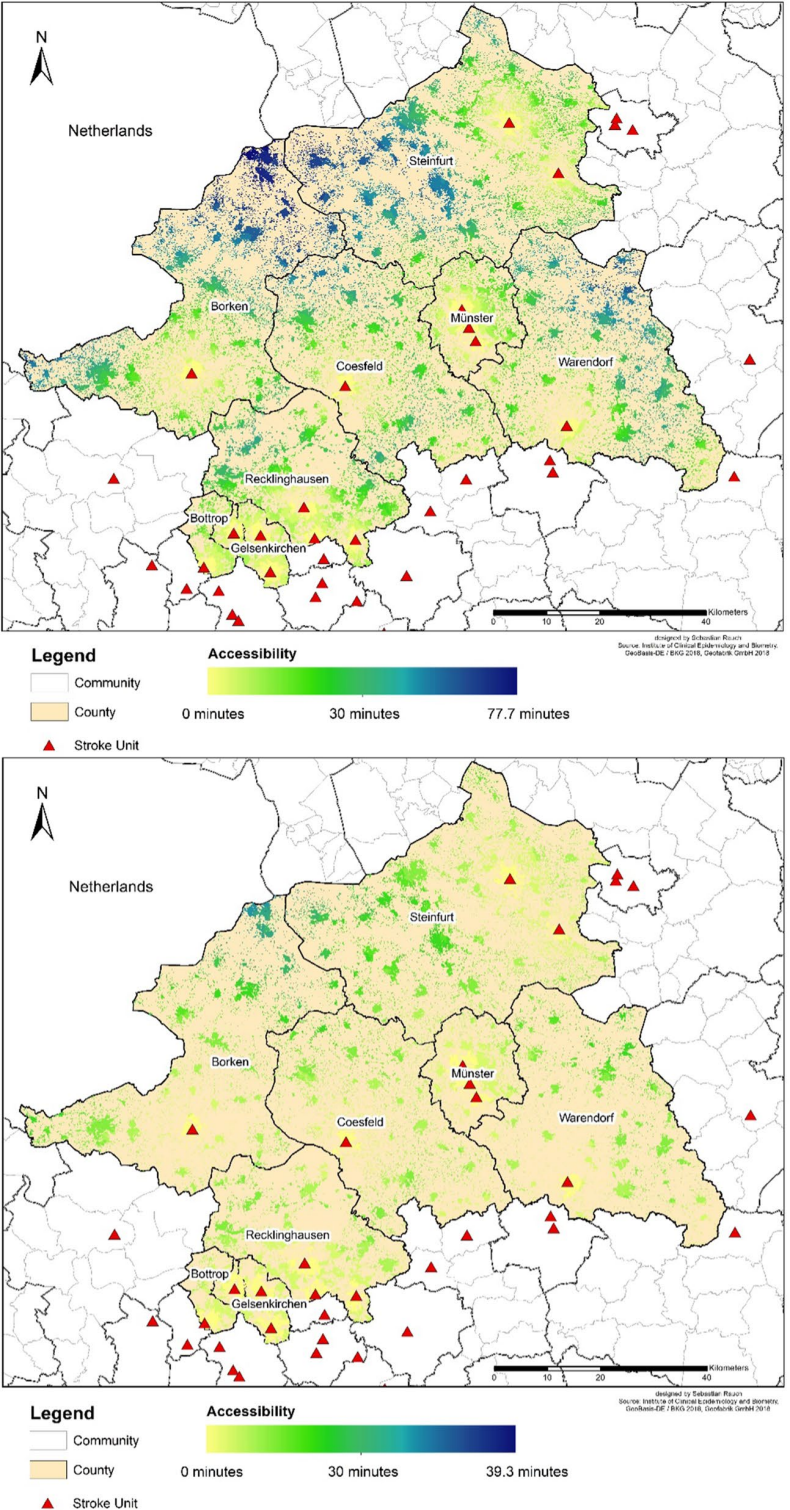
Accessibility of stroke units: Worst-case scenario (M30) by day (top) and best-case scenario (P40) by night (bottom)

A comparison between expected traffic peaks during rush hours by day (M30) and more relaxed situations at night (P40) shows a significant difference (Fig. 7). 39.8% of the people cannot be reached within 20-min driving time (M30) during the day, while comparatively few of 8.6% cannot be accessed in the P40 scenario. The maximum driving time during the day is 78.5 min (M30), during the night only 39.3 min (P40). In particular, this difference shows that the ability to drive fast and therefor reduce the journey-, but also the transport-period is crucial. It also shows different traffic situations can have a decisive influence on the driving time of ambulances.

Despite the fact, that the median travel time in that scenario (M30) is slightly worse over day, due to the population distribution (workplaces, leisure activities etc.), the number of potential patients is lower.

**Fig. 8:**
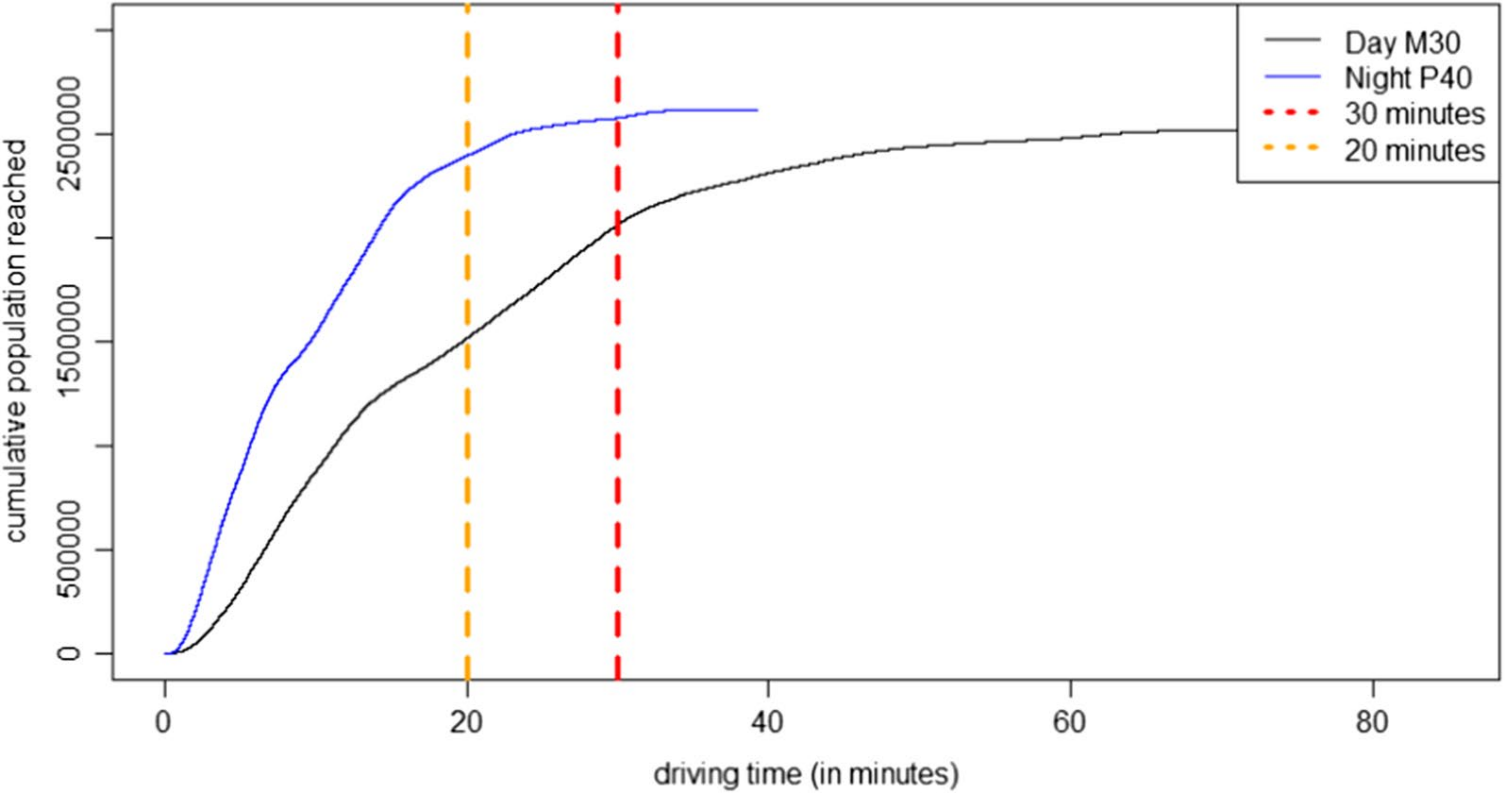
Population by driving time to the nearest stroke unit: Worst-case scenario (M30) by day and best-case scenario (P40) by night

To highlight the influence of the overall traffic situation, Fig. 8 shows the difference between the worst-case (M30) during the day and best-case scenario (P40) during the night. Due to dense traffic or traffic jams especially in urban regions during the day and the rush hours, the worst-case scenario is a realistic assumption while by night the traffic situation is less stressed, because no traffic jam to this level is expected at this time.

### Focusing different spatial categories

Overall, the marginally better accessibility during daytime can also be explained by workplace agglomerations outside of city centers. However, these agglomerations are more likely to be found in urban areas than in less densely populated regions. The median accessibility (base scenario) of 7.2 min (day) and 6.9 min (night) in urban areas is significantly lower than those in less urbanized regions with a lower population density (16.3 min (day); 15.8 min (night)) (Tables 3 & 4).

**Table 3:** Results of the accessibility analyses in the region of Muenster by day and type of region

	Quartile 1	Median	Quartile 3	Maximum		Share of people not reached within 30 minutes	Share of people not reached within 20 minutes
Rural–urban transition							
Base	11.2	16.3	21.9	54.9		6.5	32.0
M30	16.1	23.3	31.4	78.5		17.6	55.2
P40	8.0	11.7	15.7	39.3		1.5	13.0
Urban areas							
Base	4.9	7.2	10.2	25.8		0	2.2
M30	6.9	10.3	14.6	36.8		1.4	13.2
P40	3.5	5.1	7.3	18.4		0	0

**Table 4:** Results of the accessibility analyses in the region of Muenster by night and type of region

	Quartile 1	Median	Quartile 3	Maximum	Share of people not reached within 30 minutes		Share of people not reached within 20 minutes
Rural–urban transition							
Base	10.9	15.8	21.1	54.9	10.9		33.3
M30	15.5	22.5	30.2	78.5	28.9		57.9
P40	7.8	11.3	15.1	39.3	2.7		13.9
Urban areas							
Base	4.7	6.9	9.9	24.4	0		0
M30	6.7	9.9	14.2	34.9	1.3		13.9
P40	3.3	4.9	7.1	17.4	0		0

These results reflect the location policy of mostly very central locations of the stroke units. In urban districts, both, day and night populations are reached within 30 min in the base scenario. While in the best case this applies also to both examined points in time, at nighttime already 2.2% of the population is not reached within 20 min. With significantly reduced driving speed, less densely populated regions show worse supply situation. Even in the best-case scenario (P40), 2.7% of the population are not reached within 30 min. Assuming a very dense traffic situation (M30), over 50% of the population, both during day and night, cannot be accessed within 20 min for a potential first care.

## Discussion

An essential human right is the “right to health”, which includes the concept of equality of living conditions [62, 64]. However, we show in this study that various locations of stay can influence the risk of receiving adequate help quickly in the event of a stroke. Even though we found accessibility to stroke units overall can be considered good, for the majority of the population inequalities are evident in space and time.

By using spatial-quantitative methods and GIS the central results confirm that space has a decisive influence on personal health risk. Previous accessibility analyses in emergency care mostly use residential populations to estimate supply potentials. Thus, they assume the applied nighttime scenarios as used in our study, but disregard spatiotemporal variations in where people stay. By using state-of-the-art macroscale accessibility-methods and a very detailed time-dependent population distribution, it is possible to make more accurate estimates of the temporal accessibility of the entire population than only for fixed catchment areas. With it we document that risk is variable distributed over the course of a day in space.

However, the approach has some limitations due to the data and its accuracy. In general, we assume, that in case of an emergency the closest facility will be chosen [42, 46, 59, 60]. We are aware that this assumption is not always true in reality. Anyhow, the presented type of accessibility analysis is considered an objective, location-based approach [17, 25] and specific spatial insight is possible due to the strongly disaggregated population data. Of course, the approach includes errors due to the spatial and thematic highly resolved population disaggregation. Nevertheless, the validation results show that this approach makes it possible to estimate the spatiotemporal distribution of the population with high accuracy. With respect to population data, 0.3% of the day- and 0.2% of the nighttime population was not considered in our results due to topological errors within the network.

Evident human dynamics linked with e.g. leisure and consumer behavior or the partial temporal overlap of the defined daytime (7 a.m. to 7p.m.) with the real school hours besides other aspects introduce still generalized assumptions in our assessment. Nevertheless, the low MAE values testify to the high accuracy under these given data settings and we believe our estimates feature even higher accuracies when using the LAU input data.

In our experiment, the nighttime shows a slightly worse health supply coverage. This can be attributed to the concentration of jobs in central locations and thus rather close to health care facilities. Because a centrally oriented planning of stroke units, also in general health care planning, focus mainly on urban areas with both, many residential locations and workplaces in the immediate vicinity. Due to the slightly higher numbers of people who have no access within 20 min at night, we assume that with the increasing centralization of hospital locations, rural residents in particular will face a worse accessibility situation in the future.

The centralization of hospital locations certainly contributes to quality assurance and perhaps even improvement. However, this also leads to a significant deterioration of the accessibility situation, especially in less densely populated peripherally located areas, where first aid for stroke patients is becoming increasingly important due to aging populations. According to the current population forecast 2040 of the BBSR [8], the demographic aging in Germany will continue (average age: 2017: 44.3 years; 2040: 45.9 years). Centrally located (urban) regions show a significantly more favorable development until 2040 (average age 2017: 43.4 years; 2040: 44.4 years) than peripherally located regions (2017: 47.3 years; 2040: 50.3 years) (BBSR [8]: 4–5).

This analysis allows to reveal the insufficient care capabilities in peripheral locations. Since stroke units can hardly be operated there for economic reasons, new technics are needed to treat patients in a specialized, remote manner using telemedicine [31]. Another promising concept is the use of Mobile Stroke Units (MSU). These vehicles provide a prehospital care using tools for diagnosis and treatment of a stroke and are therefore a valuable instrument to rural areas where patients face worse access to stationed stroke care (Kunz et al. [32], Mathur et al. [40]: 1). Especially for remote region, Air-Mobile Stroke Unit approach is also promising [63].

## Conclusion

In general, we reveal inequivalence of the risks in case of a stroke depending on locations and times of the day. The ability to drive at high average speeds is a crucial factor in emergency care. This is the only way to ensure that patients receive the right initial treatment in time. Thus, accessibility is not only an important criterion for decision-making by health professionals and policy makers, measures of accessibility (like travel-time to the next hospital) also offers individuals the opportunity to review their care situation. In addition, high-resolution spatial population data is an elementary component of care analyses and spatial epidemiology. The ability to clearly locate specific population groups provides the opportunity to identify risk areas in preventive research. The effects of different population distributions reinforce previous findings on inequality in medical care [28, 53]. Thus, we show that a multitemporal view of the population distribution can produce variations in the accessibility situation in the same way as the use of different speed scenarios. Therefore, a static population distribution always requires an adapted speed scenario to be chosen depending on the time of investigation. Even if this approach cannot exactly describe individual paths in reality, it creates a picture that is closer to actual practice.

In methodological terms it has been displayed, that the combination of heterogeneous free data sets from censuses or OSM enables to map reality in ever better spatial and temporal resolution. The combination of spatially high-resolution population data and different speed scenarios opens up numerous benefits for the planning process. The method is applicable for strokes, but also for other emergency events or even general care analyses when using a car.

## Supplementary Information

**Additional file 1.** The map shows the modeled population distribution during the day in high resolution.

**Additional file 2.** The map shows the modeled population distribution during the night in high resolution.

## Data Availability

The accessibility network is based on Open Street Map data and is therefore open access. The modeled population distribution was created by DLR and is therefore not publicly available.

## Footnotes

1 classified by the German Federal Institute for Research on Building, Urban Affairs and Spatial Development [7]: *City regions*: Regions in which at least 50% of the population lives in large and medium-sized cities and in which a large city with a population of 500,000 or more is located, as well as regions with a population density of at least 300 inhabitants per km^2^, not including large cities. *Regions with urbanization approach*: Regions in which at least 33% of the population lives in large and medium-sized cities with a population density of between 150 and 300 inhabitants per km^2^ and regions in which at least one large city (100.000 and more inhabitants) is located and which have a population density of at least 100 inhabitants per km^2^ excluding large cities. Further we will use the term “rural–urban transition” for these regions.
