# Supplementary figures and images for: Risk and space: modelling the accessibility of stroke centers using day- & nighttime population distribution and different transportation scenarios
Source: Int J Health Geogr. 2021 Jun 29;20:31. doi: 10.1186/s12942-021-00284-y (PMC8243862; doi:10.1186/s12942-021-00284-y)

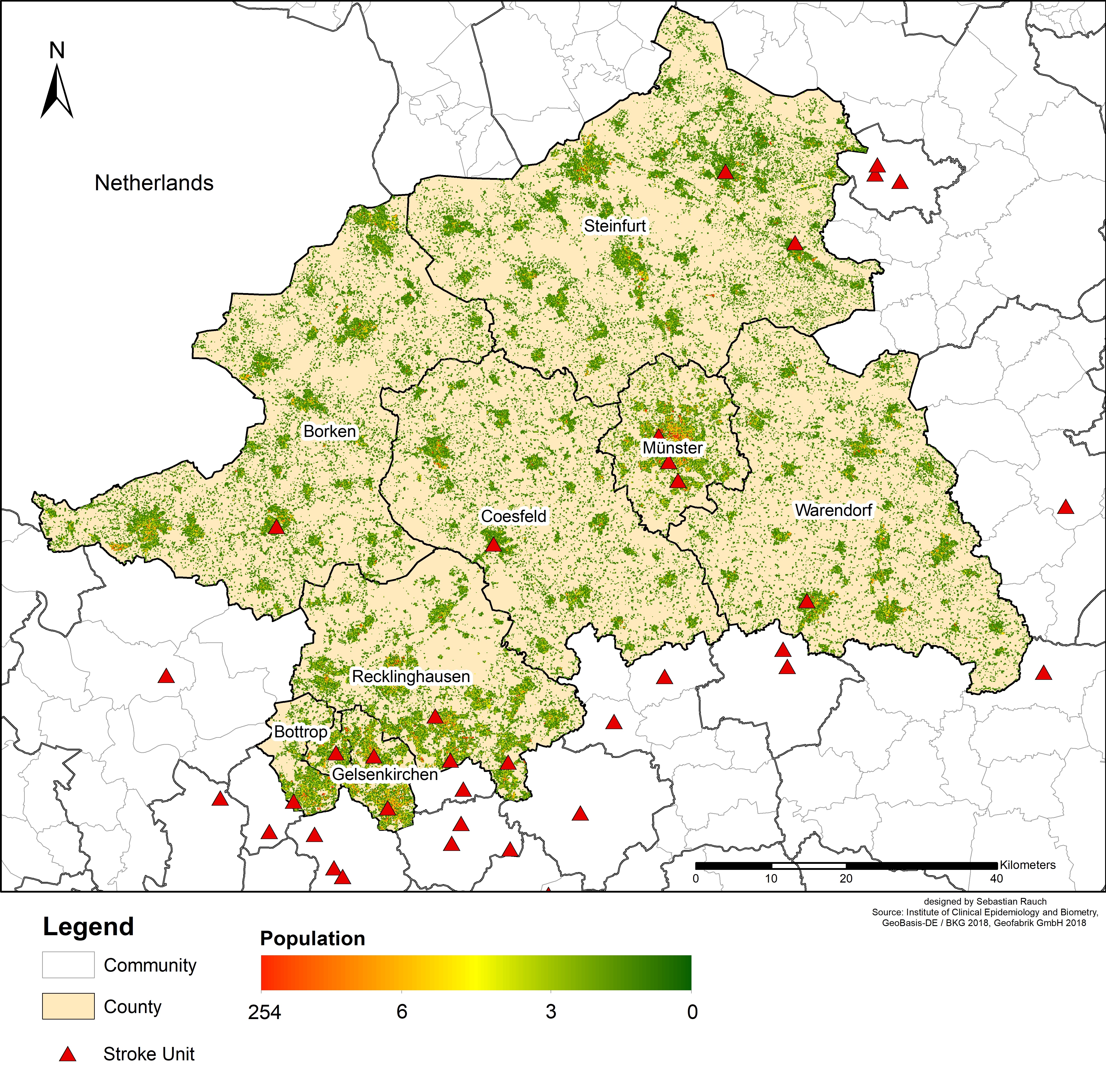

Supplement: Supplementary file 1 — Additional file 1. The map shows the modeled population distribution during the day in high resolution. [file 12942_2021_284_MOESM1_ESM.jpg]

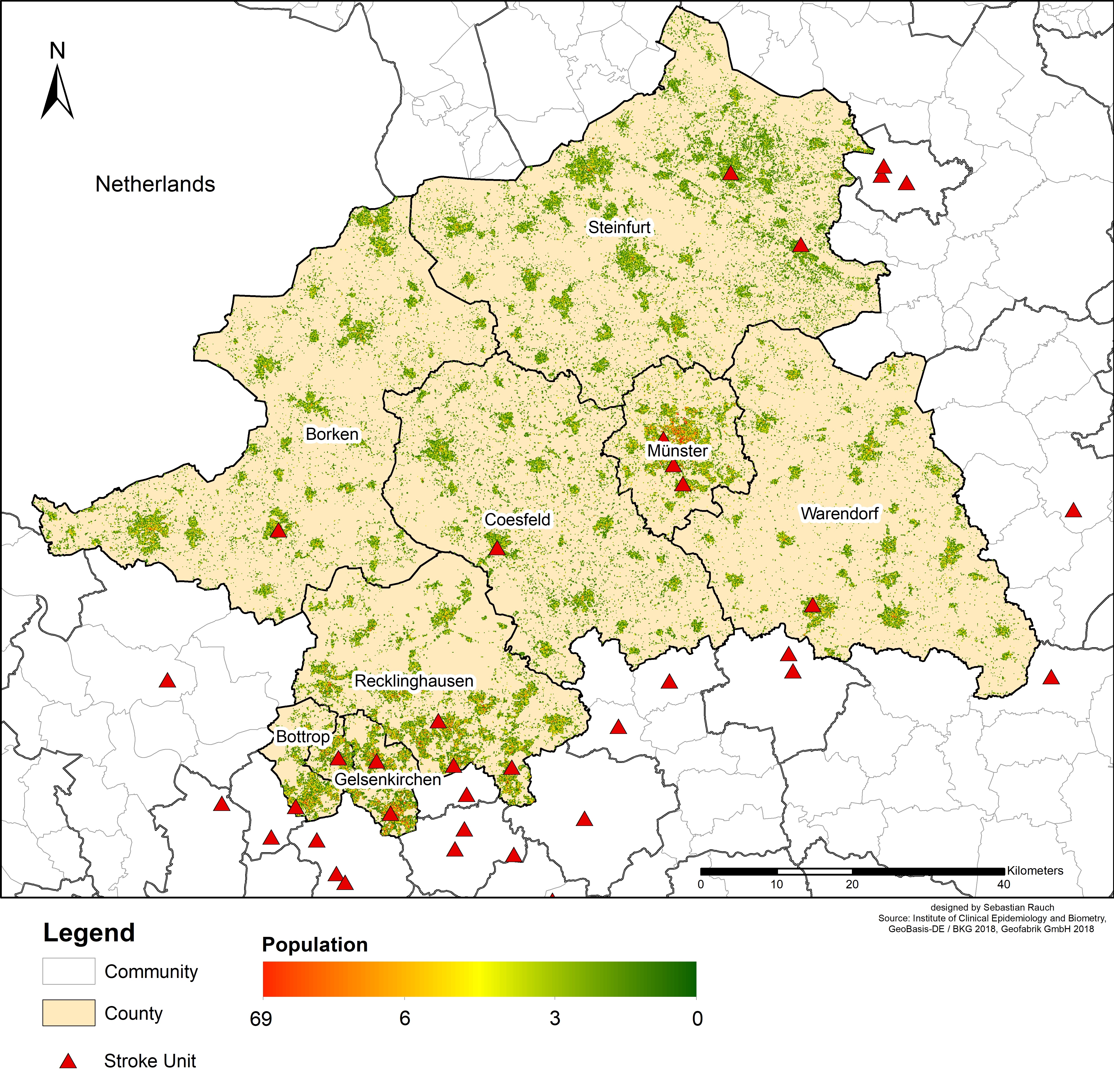

Supplement: Supplementary file 2 — Additional file 2. The map shows the modeled population distribution during the night in high resolution. [file 12942_2021_284_MOESM2_ESM.jpg]
